# Predictors of concurrent heroin use among patients on opioid maintenance treatment in France: a multilevel study over 11 years

**DOI:** 10.1186/s12954-024-00934-x

**Published:** 2024-01-19

**Authors:** Eric Janssen, Mike Vuolo, Stanislas Spilka, Guillaume Airagnes

**Affiliations:** 1 French Monitoring Centre for Drugs and Drug Addiction (Observatoire Français des Drogues et des Tendances Addictives - OFDT), 69 rue de Varenne, 75007 Paris, France; 2 Department of Sociology, Ohio State University, 238 Townhsend Hall, 1885 Neil Avenue Mall, Columbus, OH 43210 USA; 3 General Population Surveys Unit, Research Centre on Population Epidemiology and Health (Centre de Recherche en Epidémiologie et Santé des Populations—CESP), Unit 1018, INSERM, Villejuif, France; 4 UFR de Médecine, Faculté de Santé, AP-HP, Centre-Université Paris Cité, 20 rue Leblanc, 75015 Paris, France; 5 Population-Based Cohorts Unit, UMS 011, INSERM, 16 avenue Paul Vaillant-Couturier, 94800 Villejuif, France

**Keywords:** France, Heroin, Heterogeneity, Multilevel analysis, Opioid maintenance treatment

## Abstract

**Background:**

Consistent reports from health professionals suggest that heroin is commonly used by patients undergoing opioid maintenance treatment (OMT) in France, potentially jeopardizing their recovery process. However, there has been no formal epidemiological assessment on the matter.

**Methods:**

We use a yearly updated compendium retrieving information on patients admitted in treatment centres in France between 2010 and 2020. Given the hierarchical nature of the data collection, we conduct 2-level modified Poisson regressions to estimate the risks of past month heroin use among patients on OMT.

**Results:**

Despite an overall decreasing trend over time, heroin use among patients on OMT is indeed common, with half of patients declaring concurrent use. Our study unveils differentiated risks of heroin use vary according to the type of OMT, with patients on methadone more likely to use heroin compared to those on buprenorphine. The use of multilevel-related measures also uncovers high heterogeneity among patients’ profiles, reflecting different stages in the treatment process, as well as differentiated practices across treatment centres.

**Conclusion:**

Opioid maintenance treatment is associated with heroin use, in particular when methadone is involved. The heterogeneity among patients on OMT should be given particular attention, as it underscores the need for tailored interventions.

## Introduction

Opioid maintenance treatment (OMT, sometimes referred to as opioid substitution therapy, opioid replacement therapy or medication-assisted treatment) is widely accepted as one of the most effective interventions for opioid use disorder (OUD), with positive health outcomes and improvement in a vast array of quality-of-life dimensions [1, 2]. The introduction of methadone to the public in 1947, followed by the pioneer experiments of Dole, Kreek and Nyswander [3, 4], induced a shift in handling and managing OUD as well as in perceptions among people who use drugs (PWUD): it acknowledged the biological nature of opioid addiction, the severe withdrawal symptoms, the withdrawal-induced suffering and established need for medical support to manage craving that may influence the use of heroin (e.g. ‘willing is not enough’). Complementarily, buprenorphine was approved for medical use in the US in 1981 and is now commonly prescribed both in emergency settings [5] or by primary care providers [6]. Comparing the effectiveness of methadone versus buprenorphine yields inconclusive results: while studies argue the superiority of methadone in improving misuse outcomes in opioid patients (decreased risk of relapse, improvement of psychological status [7]), buprenorphine reduces risk of overdose and is more easily manageable on a daily basis [8]. Although a safer agent—according to the CDC, one-third of all prescription drug-related deaths in 2012 were related to methadone overdose—and despite its effectiveness, buprenorphine remains underused in treating opioid use disorder [9].

Despite the aforementioned virtues, OMT in France has been the subject of controversy, with consequent delay in their implementation. Both methadone and buprenorphine are recommended by the relevant agencies for detoxification from heroin and for opioid maintenance therapy. The State-driven, universal social security coverage has provided facilitated access to OMT [10, 11], with special attention towards PWUD with the fewest resources. However, methadone was officially introduced as part of a State endorsed harm reduction policy in 1995 only, that is, several decades after the implementation of methadone in the USA and with considerable delay as compared to other Western European countries [12]. In order to provide a better response to the rapid spreading of HIV, buprenorphine was authorized in 1996 within a relaxed regulatory environment: buprenorphine can be prescribed by any general physicians, regardless of their training in addictions or lack thereof. In contrast, methadone maintenance treatment must still be initiated within a hospital/treatment centre. Alternative medicated procedures, such as morphine sulphate or codeine, are not legally labelled as OMT. Since 2002, however, morphine sulphate can be prescribed in case of intolerance to methadone and buprenorphine, but under strict regulations. To date, morphine sulphate is marginally prescribed by a small number of outreach field general practitioners attending elderly people who use opioids (PWUO). In a similar fashion, naloxone is not regarded as a complete OMT per se, as its theoretical benefits for this purpose [13] have not been supported in clinical practice. Naloxone and combined buprenorphine/naloxone are limited to prevent intravenous injection.

Another characteristic of the French case worth attention lies on the prevalence of heroin as the main misused opioid. Contrary to the USA [14], there has been no significant rise in unprescribed opioid pharmaceuticals in France during the past decades. Misuse of opioids such as fentanyl, hydrocodone or oxycodone IS restricted to a very limited number of PWUD. Heroin has remained the most common, available and accessible illicit opioid in France, with increased diffusion in remote areas [15, 16]. As such, heroin constitutes the vast majority of opioid treatment demand. However, heroin discontinuation is not automatically induced by the medically supervised intake of OMT. Concurrent use is a common feature, in particular at early stages of treatment, increasing the odds of relapse [17–19].

In a context of resurgent heroin use in Western countries [20] and high availability [21], referring patients to OMT is a logical step to ensure heroin cessation and improve the patients’ quality of life. However, this medical, cessation-centred standpoint may be challenged by patients, who may question the benefit of an OMT as compared to the drug being treated [22]. Moreover, the severe physiology and psychological effects, including craving and withdrawal symptoms, frequently associated with the earlier steps of heroin discontinuation [23, 24], are likely to enhance the concurrent use of heroin, putting in jeopardy the cessation process and perpetuating the likelihood for negative outcomes, such as fatal and non-fatal overdoses. Despite these concerns, very little is known on concurrent use of heroin among patients on OMT in France, and even less is known when it comes to differentiating by OMT.

Thus, the objectives of this study are twofold: first, to assess the (yet) unknown prevalence of heroin concurrent use and its implications; second, to examine factors associated with heroin use among outpatients on OMT between 2010 and 2020. Adopting a repeated cross-sectional design allows us to uncover potential changes over the course of the past decade.

## Methods

### Data

The data come from a yearly updated compendium on addictions and treatments (*Recueil Commun sur les Addictions et les Prises en charge*——RECAP), carried out at the national level in France. Treatment centres in France are publicly funded, medically driven entities located within each of the sub-regional administrative areas. They provide free, anonymous access to all individuals seeking treatment for addiction, both to licit and/or illicit substances, regardless of their income, professional status, gender, race or age and aim at a complete cessation of substance use. Treatment centres provide outpatient (including in-prison) and inpatient services. Both medication-assisted treatments, such as methadone maintenance and buprenorphine prescription, and psychosocial treatment are provided.

All treatment centres are requested to provide data on patients welcomed into their premises during a full calendar year, following the European protocol for registering treatment demand, one of the European Monitoring Centre on Drugs and Drug Addiction’s key indicators [25]. Each year, 80% of treatment centres on average provide data; among those providing data, 100% of patients are included. The face-to-face, standardized questionnaire includes information on individual substance use (frequency of use, route of administration, age at onset and an assessment of the severity of use), health and sociodemographic characteristics. For a given patient, the questionnaire is completed every year. Unique identifiers assure that there is no duplicate data. The survey was approved by the National Data Protection Authority (CNIL).

### Case definition

This study focuses on clients on OMT serviced in treatment centres in France between 2010 and 2020. Patients are classified as undergoing an OMT treatment if they stated having used methadone, buprenorphine and/or an alternative procedure under medical supervision (*n* = 347,608). The survey also includes questions on unsupervised use of OMT during the past 30 days; those who used OMT in the past 30 days were discarded as we cannot disentangle whether in the 30 days prior reference point, heroin use came after supervised OMT, a necessary assumption for the analysis. In order to establish that past 30-day heroin use occurred temporally after the start of supervised OMT, we exclude the small number of patients who began supervised OMT in the past 30 days (2796 patients, or 0.8% of all supervised OMT patients). We note that the results are virtually identical with such patients included. The final sample includes 344,812 outpatients with OMT under medical supervision nested in 455 treatment centres.

### Statistical analysis

The dependent variable measures whether a patient used heroin during the past 30 days. At the individual level, the models account for the type of OMT (buprenorphine as reference, methadone, other medication) and the duration elapsed since beginning of treatment (1 year or less; 2–4 years; 5 years or more as reference). Control variables include year of survey (2010 as reference), gender (male as reference), age (15–24, 25–34 and 35–64 as reference), employment status (inactive as reference; unemployed; currently working)housing (homeless/temporary accommodation; stable accommodation as reference) and geographical location (Paris metropolitan region vs rest of the country as reference). Other substance-related questions include the use of both licit (tobacco, alcohol), unprescribed psychotropics and illicit substances (cannabis, stimulants, hallucinogens) during the past 30 days (no as reference vs yes). Given the hierarchical structure of the data, in which patients are nested in treatment centres, we estimated the relative risks and identified factors related to heroin use during the past 30 days by means of 2-level Poisson regressions with robust standard errors [26] using Stata® 17.1. The significance level for tests was set at *p* < 0.05. In order to numerically assess the clustering effect, we calculated the median rate ratio (MRR), following the proposed method of Austin and colleagues [27]. The MRR is an extension of the median odds-ratio (MOR) for count variables that translates the higher-level variance in the incidence ratio scale. Stated more practically, the MMR shows the extent to which the individual probability varies across clusters. We first display a model with main effects only. In order to assess potential changes of heroin use according to type of OMT over time, we show a second model with an interaction between these two measures. To aid interpretation, we show predicted probabilities for these two measures, obtained via the margins command in Stata.

## Results

### Descriptive statistics

The characteristics of the sample are shown in Table 1. The data reveal a stable number of outpatients following an OMT protocol over the past decade, alongside a decreasing trend in heroin use. Although an encouraging trend, the proportion of people who use heroin (PWUH) remains high: overall, more than half of the patients stated they had used heroin during the past 30 days (57.8% in 2010 vs. 49.3% in 2020). We note the decreasing proportion of patients from the Paris metropolitan region (20.4% in 2010 vs. 14.9% in 2019), reflecting the diffusion of heroin towards remote areas during the past decade. The overall sample demonstrates a constant 1 to 5 ratio male-to-female. Patients under treatment are an ageing population [mean age (SD) = 35.4 (8.5) in 2010 vs. 41.4 (9.0) in 2020], reflecting the ageing process observed among PWUO in several Western European countries. The prescription of methadone among patients serviced in treatment centres has increased (54.5% in 2010 to 63.0% in 2020), buprenorphine decreased in a similar pattern (43.2–34.1%) whereas other medicated procedures remained marginal (less than 3% in 2020). Current employment status shows a relative improvement: in 2010, one in three patients on OMT were inactive, 28.2% were unemployed and 38.1% stated they had a job. The latter proportion has been increasing since 2013, with 41% currently working in 2020, although 30% were unemployed. We note the substantial increase in the concurrent use of licit substances (+ 15 per cent points, ranging from 27.3% in 2010 to 42.5% in 2020), the decreasing use of unprescribed psychotropics (7.3% to 5.1%, mostly benzodiazepines) and a milder increasing trend in concurrent use of other illicit substance use (30.2% in 2010 to 33.9% in 2020).

**Table 1:** Characteristics of clients under OMT treatment in treatment centres in France 2010–2020

	2010	2011	2012	2013	2014	2015	2016	2017	2018	2019	2020
N	30,468	32,122	31,301	33,477	30,722	32,526	30,140	32,895	30,445	29,875	30,841
Past month heroin use	57.8	55.8	55.7	52.2	47.3	48.0	51.1	51.7	52.4	53.1	49.3
Methadone	54.5	56.5	58.6	57.2	58.3	58.2	59.6	61.6	62.4	63.1	63.0
Buprenorphine	43.2	41.5	38.9	39.5	38.6	38.2	36.5	35.2	34.1	34.2	34.1
Other	2.3	2.0	2.5	3.3	3.1	3.7	3.9	3.2	3.5	2.7	2.9
OMT ≥ 5 years	45.4	48.3	52.2	55.9	56.4	58.6	60.2	62.6	65.3	68.0	70.2
OMT 2–4 years	27.9	27.9	27.4	24.9	24.2	23.3	22.9	22.1	21.3	19.9	18.9
OMT ≤ 1 year	26.7	23.8	20.4	19.2	19.4	18.1	17.0	15.2	13.4	12.1	10.9
Males	78.3	78.5	78.5	79.3	78.5	79.0	78.3	78.7	78.4	78.8	78.7
Females	21.7	21.5	21.5	20.8	21.5	21.0	21.7	21.3	21.6	21.2	21.3
15–24	10.0	8.7	7.8	6.3	5.5	4.4	4.0	3.5	2.9	2.4	2.3
2534	37.2	37.1	38.0	36.3	35.0	33.4	31.1	29.3	26.2	24.3	21.6
35–64	52.7	54.2	54.3	57.4	59.5	62.2	64.9	67.2	71.0	73.3	76.2
Inactive	33.6	31.0	29.7	34.2	38.6	38.3	37.2	29.9	27.8	28.9	28.8
Unemployed	28.2	31.9	33.2	31.8	25.8	24.5	27.6	31.6	33.5	31.9	30.3
Working	38.1	37.1	37.1	33.9	35.6	37.2	35.3	38.5	38.7	39.2	40.9
Homeless/temporary	26.2	26.3	25.7	20.4	26.0	25.3	23.8	24.7	24.9	26.0	25.3
Stable accommodation	73.8	73.7	74.3	79.7	74.0	74.7	76.2	75.3	75.1	74.0	74.7
Paris region	20.4	21.6	18.7	21.7	18.8	17.8	16.3	15.9	15.8	14.9	16.9
Rest of the country	79.6	78.4	81.3	78.3	81.2	82.2	83.7	84.1	84.2	85.1	83.1
Licit substances	27.2	27.8	28.0	31.8	32.3	35.5	39.3	39.9	41.7	43.5	42.5
Psychotropics	7.3	6.8	7.8	7.8	6.1	5.5	5.8	6.1	5.9	5.7	5.1
Other illicit substances	30.2	28.2	30.5	32.1	27.0	27.2	29.6	31.7	34.6	36.1	33.9

*Source*: RECAP survey

### Multilevel models

The results of the multivariate analysis are shown in Table 2. Model 1 includes the main effects only. Overall, the multivariate results confirm the significant decreasing trend in past month heroin use among patients under OMT protocol over time (relative to 2010, incidence ratio rate (IRR) = 0.92 with 95% confidence intervals [0.88–0.96], *p* < 0.0001 in 2013, 0.88 [0.82–0.95], *p* < 0.001 in 2017, 0.86 [0.79–0.93], *p* < 0.001 in 2020). Controlling for the other variables, heroin use among patients with OMT is associated with gender, with females showing a lesser tendency (IRR = 0.98 [0.97–0.99], *p* < 0.001). The coefficients for age show that heroin use is more common among the youngest patients: relative to those aged 35–64, the associated risks are 20% higher for those aged 15–24 (IRR = 1.20 [1.16–1.24]) and 12% for those aged 25–34 (IRR = 1.12 [1.10–1.14], *p* < 0.001 in both cases). Confirming the descriptive results, heroin use is less frequent among patients on OMT from the Paris region (IRR = 0.67 [0.58–0.78], *p* < 0.001). Heroin use is also strongly related to job status, with patients in the labour force having higher risks, either if they are unemployed or have a job (+ 11% and + 13% respectively, *p* < 0.001 in both cases), relative to patients out of the labour force. The latter tendency is confirmed by the housing variable as well: past month heroin use is less frequent among patients with no or temporary accommodations (IRR = 0.98 [0.96–1.00], *p* < 0.05). Unsurprisingly, heroin use is more common among patients under OMT for a year or less, with an increased risk of 15%, which lowers to 8% for those having been in treatment for 2–4 years compared to patients on treatment for 5 years or more (*p* < 0.001 in both cases). As compared to buprenorphine, methadone is independently associated with increasing risk of heroin use (IRR = 1.18 [1.15–1.21], *p* < 0.001), whereas it is much less common among patients on other types of OMT (IRR = 0.64 [0.51–0.80], *p* < 0.001). We also note that the use of heroin is positively associated with the use of other substances, both licit (+ 17%, *p* < 0.001) and illicit (+ 22%, *p* < 0.001). Conversely, heroin use is negatively associated with unsupervised use of psychotropics (-5%, *p* < 0.01), suggesting self-medication in order to cope with comedown-induced anxiety.

**Table 2:** Factors associated with heroin use among patients with OMT in France 2010–2020

Variables	Categories	Model 1		Model 2	
		IRR	95% CI	IRR	95% CI
Year (Ref: 2010)	2011	0.99	[0.96–1.02]	0.95*	[0.91–0.99]
	2012	0.96*	[0.93–0.99]	0.90***	[0.86–0.94]
	2013	0.92***	[0.88–0.96]	0.89***	[0.85–0.94]
	2014	0.84***	[0.78–0.92]	0.79***	[0.73–0.87]
	2015	0.86***	[0.80–0.93]	0.82***	[0.75–0.89]
	2016	0.89**	[0.83–0.96]	0.84***	[0.78–0.91]
	2017	0.88***	[0.82–0.95]	0.83***	[0.76–0.89]
	2018	0.88***	[0.83–0.94]	0.83***	[0.77–0.89]
	2019	0.90**	[0.85–0.97]	0.82***	[0.76–0.89]
	2020	0.86***	[0.79–0.93]	0.77***	[0.70–0.85]
Gender (Ref: males)	Females	0.99**	[0.97–0.99]	0.99*	[0.97–1.00]
Age	15–24	1.19***	[1.15–1.23]	1.19***	[1.15–1.23]
(Ref: 35–64 y.o.)	25–34	1.11***	[1.09–1.13]	1.11***	[1.09–1.13]
Job status	Unemployed	1.12***	[1.09–1.14]	1.12***	[1.09–1.14]
(Ref: inactive)	Working	1.13***	[1.11–1.15]	1.13***	[1.10–1.15]
Housing (Ref: stable accommodation)	Homeless/temporary accommodation	0.98*	[0.97–1.00]	0.98*	[0.97–1.00]
Geographical location (Ref: Rest of the country)	Paris region	0.67***	[0.58–0.78]	0.67***	[0.58–0.78]
OMT—type	Methadone	1.18***	[1.15–1.21]	1.08**	[1.03–1.13]
(Ref: buprenorphine)	Other medication	0.64***	[0.51–0.79]	0.60	[0.33–1.12]
Time elapsed since onset	1 year or less	1.15***	[1.12–1.18]	1.15***	[1.12–1.18]
(Ref: 5 years or more)	2–4 years	1.08***	[1.06–1.10]	1.08***	[1.06–1.10]
Other substance use	Licit substances	1.19***	[1.15–1.22]	1.19***	[1.15–1.22]
(Ref: no use)	Illicit substances	1.22***	[1.17–1.27]	1.22***	[1.17–1.27]
	Psychotropics	0.86**	[0.83–0.89]	0.86**	[0.83–0.89]
Year × OMT	2011 × methadone			1.06***	[1.03–1.10]
	2011 × Other			1.27	[0.84–1.92]
	2012 × methadone			1.12***	[1.07–1.17]
	2012 × Other			1.18	[0.67–2.05]
	2013 × methadone			1.05*	[1.00–1.10]
	2013 × Other			0.96	[0.56–1.62]
	2014 × methadone			1.10***	[1.04–1.16]
	2014 × Other			1.34	[0.85–2.10]
	2015 × methadone			1.09***	[1.04–1.16]
	2015 × Other			1.01	[0.59–1.73]
	2016 × methadone			1.10***	[1.04–1.16]
	2016 × Other			0.91	[0.54–1.53]
	2017 × methadone			1.11***	[1.05–1.17]
	2017 × Other			1.05	[0.55–1.99]
	2018 × methadone			1.11***	[1.05–1.17]
	2018 × Other			0.95	[0.50–1.81]
	2019 × methadone			1.16***	[1.09–1.23]
	2019 × Other			1.05	[0.56–1.97]
	2020 × methadone			1.19***	[1.11–1.26]
	2020 × Other			1.02	[0.58–1.80]
var(cons[centres])		1.27***	[1.20–1.34]	1.27***	[1.20–1.34]
ICC/VPC		0.25		0.25	
MMR		1.61	[1.52–1.69]	1.61	[1.52–1.69]
N		344,754		344,754	

*Source*: RECAP survey

Licit substances include alcohol, tobacco and psychotropics under medical supervision. Illicit substances include other opioids, stimulants and hallucinogens. The category ‘Psychotropics’ refers to unprescribed use. ICC/VPC: intra-correlation coefficient/variance partitioning coefficient; MRR: Median rate ratio. 58 individuals were discarded due to missing information

*p < 0.05; **p < 0.01; and ***p < 0.001

The random effect reflects significantly different averages across treatment centres and concomitantly high within-cluster homogeneity, suggesting that heterogeneity is not restricted to individual-level variability. The MRR is 1.61 with 95% confidence limits that exclude the value 1, denoting significant between-cluster variance. Note that the equivalent of the ICC/VPC for Poisson regression [27]:575] provides a similar result.

**Fig. 1:**
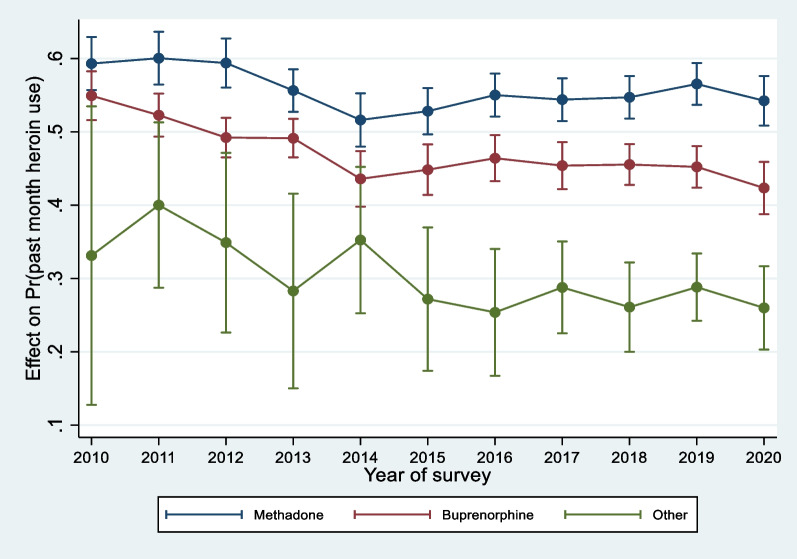
Predicted probability of past month heroin use by year and type of opioid agonist treatment, 2010–2020

In order to better assess the association of the type of OMT with past month heroin use over time, we ran a second model including an interaction. We note that the prior results hold, with a pronounced effect in the decreasing trend over time. The interaction suggests an exacerbated tendency to use heroin among patients on methadone over time, taking place smoothly at first, with a 6% increase in 2011 as compared to 2010 up to a 11% increase in 2018, then followed by a sharper increase, reaching 19% in 2020. The more erratic tendency among patients with other types of OMT is not statistically significant. For ease of interpretation, we show the interaction effect as predicted probabilities in Fig. 1, with other variables held constant at their respective means. The probability of heroin use has remained higher among patients on methadone, consistently greater than 0.5. Overall, the probabilities of past month heroin use among patients on methadone and buprenorphine lowered between 2010 and 2014, slightly increased until 2016 and have remained stable ever since, whereas an overall decreasing trend shows for patients with other type of medication. We note the associated wide confidence intervals of the latter, reflecting the low prevalence.

## Discussion

### Findings

Taking advantage of a nationwide, standardized, yearly updated dataset containing information on all PWUD under treatment in mainland France between 2010 and 2020, this study sought to identify the factors associated with the use of heroin among outpatients on OMT treatment centres for heroin. To our knowledge, this is the first study devoted to OMT patients with such a substantial sample size in France.

Our study suggests a consistent decreasing tendency in heroin use among OMT patients in France during the past decade, a tendency detected in other Western European countries [28]. This encouraging trend is somewhat tempered by an absolute figure: in 2020, almost half of OMT patients had used heroin during the past 30 days, with differentiated level of use according to the type of OMT considered. According to our results, patients on methadone maintenance treatment show an exacerbated tendency of heroin use over time as compared to patients on buprenorphine (55% vs 47%). Our results differ from what is commonly described in the literature: according to a meta-analysis [29], placebo-controlled clinical trials suggest that methadone and buprenorphine, with the latter prescribed at high, fixed doses, are equally effective at reducing opioid use and retaining patients in treatment, both in the short [30] and long term [29].

From a clinical perspective, the difference in concurrent heroin use between buprenorphine and methadone recipients can be explained by the pharmacology of these treatments. On the one hand, buprenorphine, as a partial agonist of mu-opioid receptors with high affinity, may prevent the use of other opioids during treatment because such opioids could produce only a few psychoactive effects. On the other hand, the psychoactive effects of heroin that patients seek are still present when treated with methadone. In addition, buprenorphine is often easier to abuse than methadone (e.g. by snorting or injection), so patients insufficiently stabilized by their OMT may find it easier to regain the psychoactive effects they seek by abusing buprenorphine than methadone, especially as patients are generally informed of the risk of fatal overdose when abusing methadone.

Furthermore, we hypothesize that this original finding reflects uncovered heterogeneity in patients’ profiles according to the type of OMT, unaccounted for in studies focusing on an average effect: for instance, patients on methadone may have been more exposed to negative life events [31], known to enhance substance misuse. By extension, patients on methadone are exposed to more severe OUD-related symptoms. This result mirrors those of a recent study suggesting the emergence of distinct groups of people who use fentanyl and more generally opioids [14]. Moreover, heterogeneity goes beyond sociodemographic profiles and may also reflect the wide array of steps that patients must undertake in their struggle for recovery. Alternative explanations could be variations in treatment access and provision, unmeasured frailty leading to lesser compliance with maintenance protocol, as well as differentiated practices from attending physicians [32]. This finding underlines the need for a more individualized approach in treatment.

On a complementary perspective, physicians might be reluctant to prescribe high dosages of methadone, leaving some patients under-treated. For instance, in 2019, the average dosage of methadone among treated patients was of 50 mg per day, which is considered below the therapeutic range, and might result to an increased likelihood of co-use [33]. This interpretation is supported by the use of cluster-related measures unveiling significant between-cluster heterogeneity: care provided to OMT patients is strongly centre-dependent, with some centres less likely to supply extended services such as professional psychosocial support. Our results underline the need for specific interventions rather than promoting new treatments, which are not risk-free. Interventions include building awareness among staff in the first place, since we believe the concurrent use of heroin to be underestimated by health professionals. Moreover, recent qualitative research has underlined the importance of patient viewpoints on their OMT that may considerably differ in terms of objectives and self-perception from health professionals’, a gap believed to facilitate relapses [22, 34]. Consistent findings have underlined the role of professionals’ attitude modulating patients’ success of recovery: negative feedback nurtures the high sensitivity of PWUH to stigma and undermines their capacity for self-empowerment, which in turns increases the odds of relapse [35–38]. Interestingly, recent studies have demonstrated that negative feedback has biological implications mediating the association with relapse [39].

On the other end, our results suggest a significantly lower propensity in heroin use among patients on other OMT (morphine sulphate, codeine). However, these results should not be interpreted as a protection as compared to methadone and buprenorphine. Firstly, our study did not follow an evaluation protocol that would permit such an interpretation. Secondly as previously stated in the introduction, morphine sulphate and codeine are not legally labelled as OMT in France. Moreover, they are marginally prescribed (less than 4%), and mostly to elderly people who have engaged in OMT over a long period. These stabilized patients are by definition less prone to use heroin. Hence, the observed differences in probability of use mirrors differences in profiles and trajectories of use rather than measuring a potential side effect.

The study also underscored a wide array of predictors of heroin use among OMT patients: males and younger patients were more likely to use concurrently heroin and their OMT. In line with prior studies, heroin use among patients with OMT in France is also strongly related to the concurrent use of other psychoactive substances, including alcohol, tobacco, cannabis, stimulants and/or hallucinogens. This finding is in line with the hypothesis of broader, self-reinforcing polysubstance (mis)use [17, 18]. Longer duration of OMT is significantly correlated with reduced heroin use: time elapsed since onset can be viewed as a proxy of maintenance in therapy and subsequent stabilization [40]. Concurrent heroin use is also positively associated with recent onset of OMT, a reminder that heroin discontinuation is a delayed, unstraightforward process that is difficult to achieve due to the severe discomfort induced by withdrawal symptoms. More surprisingly, the positive association persists among patients in the labour force, a trend confirmed by the higher propensity of concurrent use among those with a stable housing. These results go against prior findings showing the lack of financial support as a predictor of concurrent use, relapse and dropout [18, 41], and others underlying the positive influence of quality-of-life indicators on opioid abstinence [28]. Plausible explanations for concurrent use of heroin among patients on OMT in the labour force refer to increased purchasing power associated with stable wages, and a potentially more stressful lifestyle. On the one hand, maintaining professional activity during treatment is common procedure in order to enhance complete rehabilitation. On the other hand, personal frailty associated with poor conditions in the working environment and stigma is likely to trigger substance use. Concurrent use of heroin is part of a mechanism to cope with job-induced stress [31], as stated by the tension-reduction hypothesis [42]. Similar associations have been found with unemployment [43] and returning to work [44, 45].

### Limitations

Several limitations must be acknowledged. First, the study used treatment centre data, an advantageous approach for accessing this hard-to-reach population. However, this data source is not representative of the entire population of PWUD on OMT in France. Treatment centres are likely to underrepresent casual recreational PWUD or those who have not sought treatment or are in harm reduction facilities. Moreover, buprenorphine is commonly prescribed by general practitioners, which are not covered by the study [46]. Second, heroin use may be underestimated since patients on OMT are likely to be reluctant to disclose concomitant substance use. Third, although the study includes a wide range of predictors, the data make no reference to ethnicity. Identification for race/ethnicity used in other national contexts does not formally exist in France by both law and for the expressed purpose of preventing discrimination through categorization. In the same way, the data did not include the dose levels of methadone and buprenorphine, protected by medical confidentiality. Fourth, the distinction between methadone in syrup and methadone in capsule form was not possible because this information is not collected. However, since patients on methadone are usually initially treated with a syrup form during the first year of treatment and then with a capsule form according to the French guidelines [47], the sample of treated patients tend naturally to evolve over time in the direction of an increase in the proportion of patients treated with the capsule form. However, with 11 years of data, a balanced proportion of patients taking the syrup and capsule forms likely emerges. Fifth, although this study is based on virtually exhaustive data from all addiction treatment facilities in France, it does not rule out the possibility of sampling bias due to closures/openings of treatment centres over time. Finally, our results should *not* be interpreted as evidence of a lesser efficiency of methadone in heroin discontinuation: the study is not a randomized-controlled trial and the cross-sectional data prevents any causal interpretation. For instance, one hypothesis we discuss is the possibility of treating the most severe patients with methadone rather than buprenorphine. Moreover, the study did not account for attrition nor did it explore its cause, such as patients dropping out of treatment, transferred to a general practitioner or alternative OMT [48, 49] or being released from treatment because they were deemed stabilized.

## Conclusion

Our findings highlight the need for more detailed information on socially (re)integrated patients. Particular attention should be paid to patients under methadone protocol. The heterogeneous profiles of patients on OMT deserve better attention to provide better tailored, more efficient interventions. The results also advocate for a more comprehensive framework: the unquestionable benefits of OMT on physical and mental health and overall quality of life cannot conceal differentiated practices and attitudes of health professionals, some of them unprepared to deal with the diversity of patients’ profiles. All of the above constitute a strong call for updated best practices guidelines and ongoing training, indispensable to fully meet the evolving scope of requirements from a hard-to-reach population. Clinicians should inform their patients who may be prone to use heroin with their OMT, reinforce screening and adjust OMT dosages.

## Data Availability

The datasets generated and/or analysed during the current study are not publicly available. The data contain sensitive information which allows the identification of individuals. It is therefore protected, and access can only be granted with special permission.

## Appendix

See Table 3.

**Table 3:** Factors associated with daily heroin use and favoured route of administration among patients with OMT in France 2010–2020

Variables	Categories	Daily use		Injected		Smoked		Snorted		Other	
		IRR	95% CI	IRR	95% CI	IRR	95% CI	IRR	95% CI	IRR	95% CI
Year	2011	1.11	[1.00–1.24]	0.89*	[0.80–0.98]	0.97	[0.89–1.07]	1.00	[0.95–1.05]	0.36***	[0.27–0.48]
	2012	0.98	[0.73–1.31]	0.82***	[0.74–0.90]	0.83**	[0.74–0.94]	0.92**	[0.87–0.98]	0.47***	[0.37–0.60]
	2013	0.91	[0.67–1.24]	0.76***	[0.69–0.85]	0.86*	[0.75–0.98]	0.86***	[0.80–0.92]	1.13	[0.92–1.38]
	2014	1.27**	[1.07–1.51]	0.62***	[0.55–0.71]	0.70***	[0.58–0.83]	0.78***	[0.69–0.87]	0.89	[0.67–1.17]
	2015	1.26*	[1.04–1.53]	0.66***	[0.58–0.75]	0.77*	[0.63–0.94]	0.77***	[0.69–0.86]	1.03	[0.74–1.42]
	2016	1.29*	[1.04–1.59]	0.64***	[0.56–0.73]	0.94	[0.78–1.13]	0.82***	[0.74–0.90]	0.68**	[0.51–0.90]
	2017	1.25*	[1.01–1.56]	0.62***	[0.54–0.72]	0.86	[0.71–1.04]	0.79***	[0.71–0.88]	0.71*	[0.54–0.94]
	2018	1.38*	[1.07–1.79]	0.61***	[0.53–0.70]	0.92	[0.76–1.11]	0.78***	[0.70–0.87]	0.70*	[0.52–0.92]
	2019	1.46**	[1.12–1.90]	0.54***	[0.46–0.63]	0.94	[0.75–1.17]	0.78***	[0.71–0.86]	0.84	[0.62–1.14]
	2020	1.22	[0.94–1.58]	0.47***	[0.39–0.57]	0.86	[0.68–1.08]	0.73***	[0.65–0.83]	0.92	[0.69–1.23]
Gender	Females	0.86***	[0.81–0.91]	0.87***	[0.83–0.92]	0.98	[0.93–1.04]	1.07***	[1.04–1.10]	0.99	[0.91–1.07]
Age	15–24	1.00	[0.87–1.14]	0.98	[0.88–1.10]	1.46***	[1.30–1.64]	1.38***	[1.31–1.45]	0.93	[0.80–1.08]
	25–34	1.01	[0.93–1.10]	0.89***	[0.83–0.95]	1.30***	[1.19–1.42]	1.23***	[1.19–1.26]	1.00	[0.93–1.08]
Job status	Unemployed	1.08	[0.99–1.18]	0.95	[0.89–1.01]	1.17***	[1.08–1.27]	1.25***	[1.21–1.29]	1.07	[0.96–1.19]
	Working	0.87***	[0.80–0.95]	0.83***	[0.78–0.90]	1.29***	[1.20–1.39]	1.40***	[1.35–1.45]	0.98	[0.88–1.09]
Housing	Homeless/temporary accommodation	0.91**	[0.86–0.97]	0.87***	[0.82–0.91]	0.97	[0.92–1.02]	1.09***	[1.06–1.12]	0.79***	[0.72–0.86]
	Paris metropolitan region	0.85	[0.62–1.18]	1.10	[0.90–1.36]	0.85	[0.67–1.07]	0.58***	[0.46–0.72]	0.85	[0.59–1.24]
OST—type	Methadone	0.96	[0.82–1.12]	1.46***	[1.30–1.63]	1.02	[0.88–1.18]	0.97	[0.91–1.03]	1.42***	[1.22–1.65]
	Other type	0.62	[0.31–1.24]	0.97	[0.47–1.97]	0.72	[0.39–1.31]	0.45	[0.20–1.02]	0.85	[0.42–1.71]
OST—time elapsed since onset	1 year or less	1.09**	[1.03–1.14]	0.92***	[0.88–0.96]	1.17***	[1.12–1.23]	1.14***	[1.11–1.18]	0.96	[0.88–1.04]
	2–4 years	1.13**	[1.05–1.21]	0.93*	[0.87–0.99]	1.30***	[1.21–1.39]	1.26***	[1.20–1.31]	1.04	[0.93–1.15]
Licit substances	Yes	1.02	[0.94–1.12]	1.29***	[1.22–1.37]	1.17**	[1.05–1.30]	1.27***	[1.21–1.34]	0.84*	[0.74–0.96]
Illicit substances	Yes	2.43***	[2.19–2.69]	1.38***	[1.28–1.49]	0.98	[0.90–1.07]	0.98	[0.93–1.04]	0.53***	[0.46–0.60]
Psychotropics	Yes	1.05	[0.91–1.22]	1.07	[0.97–1.18]	0.75***	[0.66–0.85]	0.71***	[0.66–0.77]	1.00	[0.84–1.18]
Year × OMT	2011 × methadone	0.92	[0.80–1.06]	1.11*	[1.01–1.23]	1.10	[0.93–1.30]	1.04	[0.99–1.10]	2.08***	[1.60–2.72]
	2011 × Other	1.05	[0.55–2.00]	1.21	[0.76–1.92]	1.34	[0.86–2.07]	1.38	[0.83–2.32]	0.59	[0.22–1.59]
	2012 × methadone	0.78	[0.59–1.02]	1.08	[0.97–1.21]	1.46*	[1.04–2.04]	1.13***	[1.05–1.21]	1.51*	[1.05–2.18]
	2012 × Other	0.91	[0.37–2.23]	1.25	[0.71–2.23]	0.79	[0.35–1.80]	1.41	[0.74–2.71]	1.17	[0.47–2.91]
	2013 × methadone	0.85	[0.66–1.11]	1.04	[0.93–1.16]	1.09	[0.95–1.27]	1.12**	[1.04–1.20]	0.96	[0.79–1.16]
	2013 × Other	1.16	[0.43–3.09]	0.97	[0.54–1.74]	0.66	[0.30–1.47]	1.27	[0.78–2.06]	0.25	[0.05–1.31]
	2014 × methadone	1.01	[0.85–1.19]	1.10	[0.97–1.25]	1.28**	[1.10–1.49]	1.15**	[1.06–1.25]	1.05	[0.87–1.28]
	2014 × Other	0.90	[0.40–2.00]	1.32	[0.71–2.47]	1.06	[0.53–2.10]	1.69	[0.99–2.91]	0.93	[0.41–2.09]
	2015 × methadone	0.96	[0.81–1.14]	1.04	[0.91–1.19]	1.23*	[1.04–1.45]	1.22***	[1.12–1.32]	0.93	[0.76–1.14]
	2015 × Other	1.07	[0.50–2.28]	0.85	[0.44–1.62]	0.98	[0.47–2.04]	1.15	[0.54–2.45]	0.50	[0.21–1.21]
	2016 × methadone	0.92	[0.77–1.10]	1.08	[0.93–1.26]	1.16	[0.98–1.38]	1.25***	[1.15–1.35]	1.01	[0.82–1.25]
	2016 × Other	0.92	[0.44–1.92]	0.94	[0.51–1.73]	0.86	[0.43–1.68]	0.92	[0.43–1.98]	0.58	[0.24–1.41]
	2017 × methadone	0.91	[0.77–1.09]	1.04	[0.88–1.23]	1.35***	[1.14–1.60]	1.26***	[1.16–1.37]	0.97	[0.79–1.19]
	2017 × Other	1.08	[0.50–2.36]	0.79	[0.37–1.69]	0.99	[0.49–1.98]	1.24	[0.52–2.98]	0.64	[0.23–1.80]
	2018 × methadone	0.98	[0.83–1.17]	1.01	[0.87–1.18]	1.36***	[1.15–1.60]	1.24***	[1.14–1.35]	0.90	[0.72–1.12]
	2018 × Other	1.08	[0.49–2.37]	0.96	[0.43–2.11]	0.86	[0.41–1.79]	0.99	[0.40–2.44]	0.44	[0.15–1.28]
	2019 × methadone	0.98	[0.83–1.17]	1.10	[0.93–1.30]	1.35***	[1.13–1.61]	1.32***	[1.22–1.44]	0.77*	[0.61–0.98]
	2019 × Other	1.43	[0.68–3.00]	1.07	[0.49–2.35]	0.75	[0.36–1.56]	1.02	[0.43–2.44]	0.29*	[0.09–0.96]
	2020 × methadone	1.11	[0.92–1.35]	1.09	[0.92–1.30]	1.46***	[1.23–1.75]	1.34***	[1.23–1.46]	0.92	[0.71–1.18]
	2020 × Other	1.49	[0.70–3.15]	1.07	[0.50–2.29]	0.72	[0.30–1.73]	1.03	[0.45–2.33]	0.69	[0.29–1.62]
	var(_cons[idstr])	2.51***	[2.10–2.99]	1.68***	[1.52–1.85]	2.40***	[2.03–2.83]	1.74***	[1.57–1.93]	3.75***	[2.95–4.77]
	N	344,754		344,754		344,754		344,754		344,754	

*Source*: RECAP survey

Licit substances include alcohol, tobacco and psychotropics under medical supervision. Illicit substances include other opioids, stimulants and hallucinogens. The category ‘Psychotropics’ refers to unprescribed use. 58 individuals were discarded due to missing information

**p* < 0.05; ***p* < 0.01; and ****p* < 0.001

## Abbreviations

CDC: Centers for Disease Control and Prevention
CNIL: Commission Nationale Informatique et Liberté (National Data Protection Authority)
IRR: Incidence ratio rate
ICC: Intra-correlation coefficient
MMR: Median rate ratio
OUD: Opioid use disorder
OMT: Opioid maintenance treatment
PWUD: People who use drugs
PWUH: People who use heroin
PWUO: People who use opioids
RECAP: Recueil Commun sur les Addictions et les Prises en charge (Compendium on addictions and treatment)
VPC: Variance partitioning coefficient
